# Environmental Drivers and Long-Term Dynamics of Copepod Communities in the Black Sea: Contrasts Between Warm and Cold Periods

**DOI:** 10.3390/biology15020184

**Published:** 2026-01-19

**Authors:** George-Emanuel Harcota, Elena Bisinicu, Luminita Lazar, Florin Timofte, Geta Rîșnoveanu

**Affiliations:** 1 Doctoral School of Ecology and Sustainability, University of Bucharest, 050663 Bucharest, Romania; geta.risnoveanu@g.unibuc.ro; 2 National Institute for Marine Research and Development “Grigore Antipa”, 300 Mamaia Blvd., 900581 Constanta, Romania; llazar@alpha.rmri.ro (L.L.); ftimofte@alpha.rmri.ro (F.T.)

**Keywords:** copepods, zooplankton, Black Sea, long term trends, environmental variables, nutrients, salinity, climate change, marine biodiversity, ecosystem monitoring

## Abstract

Copepods are key components of marine food webs, linking primary producers such as microalgae to higher trophic levels, including many fish species. This study investigates long-term changes in the composition, density, and biomass of copepod communities along the Romanian coast of the Black Sea over six decades (1956–2015), based on historical records and recent monitoring from 18 sampling stations. Mean copepod density declined markedly over the study period, particularly during the cold season, decreasing from values exceeding 1000 ind/m^3^ in the 1960s to <300 ind/m^3^ after 2000, while biomass showed weaker but comparable long-term fluctuations. Seasonal variability was pronounced, with significantly higher densities and biomass during the warm season. Generalised Additive Models (GAMs) explained up to 40–55% of the variance in copepod density and biomass, depending on the season. During the warm season, phosphate exerted a positive effect on copepod abundance, consistent with bottom-up control via phytoplankton productivity, whereas during the cold season, temperature showed a positive effect and salinity a negative effect, indicating stronger physical control of copepod persistence. Species composition shifted over time, with a reduction in constant species and an increase in rare or accidental taxa in later decades. These results indicate that climate variability and anthropogenic pressures have reshaped copepod communities, with potential consequences for food-web efficiency and ecosystem resilience in the Black Sea.

## Simple Summary

Copepods play a crucial role in marine food webs, as they feed on microalgae and are food for many fish species. The paper investigates the dynamics and the key drivers of changes in the copepod community along the Romanian coast of the Black Sea over 60 years (1956–2015). We assess seasonal and long-term changes in density, biomass, and species composition, and explore their relationships with environmental variables, including temperature, salinity, nutrients, and oxygen. The results marked decline in copepod density, biomass, and species richness starting in the early 1980s, especially during colder months Seasonal variation in species composition is evident, with distinct communities characterizing warm and cold months. These seasonal patterns are influenced not only by environmental conditions but also by shifts in the copepod community itself, as changes in the relative abundance of dominant and opportunistic species modulate overall community structure across seasons. Among environmental drivers, increased nutrient concentrations, especially phosphates and ammonium, emerged as major factors influencing copepod community structure. These findings enhance our understanding of long-term dynamics in marine zooplankton and highlight the sensitivity of copepod communities to both climatic and human-induced pressures. Moreover, they underscore the value of copepods as effective bioindicators of marine ecosystem health and provide insights to inform conservation strategies and the management of ecosystems in the Black Sea region.

## 1. Introduction

Zooplankton plays an essential role in marine ecosystems by transferring energy from primary producers to higher trophic levels, regulating biogeochemical cycles [1,2] and rapidly responding to environmental variability in temperature, salinity, nutrient availability, and pollution [3,4].

Copepods dominate zooplankton communities in terms of abundance and diversity, occupying a central position in the food web [5,6,7] as the primary link between phytoplankton and fish and larger invertebrates [8,9,10,11,12].

Their sensitivity to environmental change, combined with behavioral adaptations and physiological strategies that enable persistence under unfavorable conditions, makes them reliable indicators of water quality and ecosystem shifts [1,13].

Predator pressure and the availability of trophic resources are key variables that determine the distribution and diversity of zooplankton, directly influencing physiological processes, such as reproduction, development, respiration, and feeding [7,14,15]. In addition, temperature influences zooplankton communities by changing the quantity and quality of available food [16,17]. In eutrophic ecosystems, high levels of nitrogen and phosphorus cause the excessive proliferation of phytoplankton, favoring the appearance of “microalgae blooms” [4,18], a phenomenon that reduces water transparency, decreases the concentration of dissolved oxygen, and creates unfavorable conditions for many zooplankton species, thereby affecting biodiversity and ecosystem balance [19]. The intensity of such blooms may increase two- to threefold with a 10 °C rise in temperature [20]. Phytoplankton blooms, particularly when associated with eutrophication, typically disadvantage large, herbivorous calanoid copepods that rely on high-quality phytoplankton, such as *Calanus* spp., *Pseudocalanus elongatus*, and *Centropages* spp., due to reduced food quality, increased turbidity, and episodic hypoxia. In contrast, small-bodied, omnivorous or opportunistic copepods, such as *Oithona* spp. and *Acartia clausii*, may benefit from bloom conditions by exploiting microbial pathways, detrital food sources, and a broader prey spectrum, allowing them to persist or increase in abundance during and after bloom events [21,22].

In the current context of climate change, zooplankton respond more strongly to environmental variability, exhibiting shifts in species composition, alterations in life cycles (e.g., breeding and development), and vertical or geographical redistribution of populations, all of which can disrupt food webs and impair ecosystem functioning [22,23]. In this study, the term “more strongly” is explicitly defined to refer to a stronger response relative to other zooplankton taxa and a response exceeding the historical interannual variability of the copepod community itself. Specifically, dominant calanoid and cyclopoid copepods exhibited larger magnitude declines, clearer directional trends, and stronger statistical relationships with environmental drivers (temperature, salinity, nutrients) than typically reported for other groups, such as cladocerans or meroplankton, which often display more episodic or opportunistic dynamics. The observed long-term reductions in copepod density and biomass exceeded their natural interannual fluctuations documented in earlier decades, indicating responses beyond background variability. These patterns are consistent with previous studies demonstrating that copepods respond more predictably and sensitively to changes in temperature, stratification, and food quality, making them robust indicators of ecosystem change [24,25,26,27,28].

These dynamics place zooplankton at the forefront of marine ecological research, particularly in vulnerable regions such as the Black Sea, where the impacts of climate change are increasingly evident [10,21,22,23]. Understanding zooplankton biodiversity is essential for assessing ecosystem status and monitoring the effects of long-term environmental change [13,25] as shifts in species composition, life cycles, and distribution directly reflect the responses of these communities to climate-driven variability. This highlights the importance of zooplankton as an indicator of ecosystem health, particularly in sensitive regions such as the Black Sea.

In recent years, several national and regional studies have provided valuable insights into Black Sea plankton communities, particularly copepods. At the Romanian coast, Boicenco et al. in 2019 reported high plankton diversity (23 mesoplankton taxa identified between 2015–2017) and a good ecological status based on plankton indicators [28]. In the northwestern Ukrainian sector, Grandova et al. in 2021 recorded 30 zooplankton taxa, dominated by Copepoda and Cladocera (e.g., *Acartia* sp., *Oithona* sp., *Centropages* sp.) [29], and Kharitonova in 2023 documented significant interannual variations in zooplankton densities (e.g., 1.96 × 10^4^ ind/m^3^ in 2019 compared to 1.03 × 10^4^ in 2016 and only 1.71 × 10^3^ in 2017) [30]. On the Bulgarian coast, studies have highlighted the restructuring of zooplankton communities associated with periods of intense eutrophication and invasion of the jellyfish *Mnemiopsis leidyi* [31], while research from the Turkish sector described the seasonal distribution of ten copepod species in the Samsun area (e.g., *Calanus euxinus*, *Paracalanus parvus parvus*, *Oithona similis*) [32].

However, research on copepods in the Black Sea remains spatially and temporally fragmented, largely due to the limited availability of long-term, standardized datasets. Many studies are confined to short monitoring periods or restricted spatial coverage, such as analyses of seasonal zooplankton dynamics along the Romanian coast from 2013 to 2020 [2], investigations of mesozooplankton variability in the north-eastern Black Sea [33], and focused assessments of seasonal zooplankton composition [34]. Other work emphasizes taxonomic inventories or reviews rather than continuous time series, such as evaluations of species composition changes over time [35]. While these studies provide valuable regional insights, their limited temporal extent and spatial coverage restrict the assessment of long-term trends and basin-wide comparability, highlighting the need for multi-decadal, harmonized datasets such as the one analyzed in the present study. Moreover, comparability across regions is constrained by non-standardized methodologies, including differences in equipment, sampling depths, and timing. In addition, the relationships between biological parameters (copepods density and biomass) and environmental variables (e.g., temperature, salinity, nutrients) remain poorly understood and insufficiently documented over the long term. In the context of intensifying climate change and growing anthropogenic pressures, such as nutrient enrichment, pollution, and the introduction of invasive species, it is increasingly essential to advance our understanding of how zooplankton communities, especially copepods, respond to environmental change over long-term periods. These knowledge gaps highlight the need for integrated, standardized, and validated datasets to clarify the long-term trends in copepod communities in the Black Sea [2,36,37,38].

This paper investigates the long-term dynamics of marine copepod communities along the Romanian Black Sea coast over six decades (1956–2015). Specifically, we assess seasonal and long-term changes in density, biomass, and species composition, and analyze their relationships with key environmental variables, including temperature, salinity, nutrients, and dissolved oxygen. Our objectives are to identify major ecological trends, detect signals of climate and anthropogenic pressures, and evaluate the suitability of copepods as bioindicators for ecosystem monitoring. In this study, copepods are evaluated as bioindicators based on their ecological relevance, sensitivity to environmental drivers, and consistency of both long-term and seasonal responses. Changes in density, biomass, species composition, and spatio-temporal patterns are considered integrative indicators of climatic and anthropogenic pressures, rather than relying on a single metric. By clarifying long-term copepod responses to environmental changes, this study contributes to a deeper understanding of zooplankton dynamics in the Black Sea and supports efforts to promote the sustainable management of the marine system and resources.

## 2. Materials and Methods

### 2.1. Study Area, Sampling Collection, and Analysis

The Black Sea, a semi-enclosed sea with limited connections to the World Ocean, exhibits distinct environmental conditions that drive its complex ecological processes. One of its key characteristics is the low salinity, averaging 17–18, which is significantly lower than that of the open oceans, due to the considerable supply of fresh water from the Danube and other rivers [3]. Another fundamental feature is the permanent stratification of the water column, which separates a surface oxygenated layer (extending to 150–200 m deep) from a deep anoxic zone enriched with hydrogen sulfide (below 200–300 m). This vertical structure limits the natural mixing of the water column, reducing oxygenation of the deeper layers. As a result, hypoxic or anoxic conditions develop at depth, while nutrient accumulation in the surface layer can enhance eutrophication processes [3,25,39].

On the Romanian coast, these processes are amplified by the intense anthropogenic pressure, including nutrient inputs from the Danube River basin and associated eutrophication. Recent climate changes, in particular the warming of seawater and variations in the hydrological regime of the river, contribute to enhanced stratification and reduced oxygenation, increasing the risk of hypoxia in coastal areas [3]. Therefore, the Romanian coastal area of the Black Sea represents a vulnerable ecosystem, where the interactions between natural factors and human-induced pressures generate significant changes in the trophic networks and compromise the ecological integrity of the marine system [39].

**Figure 1:**
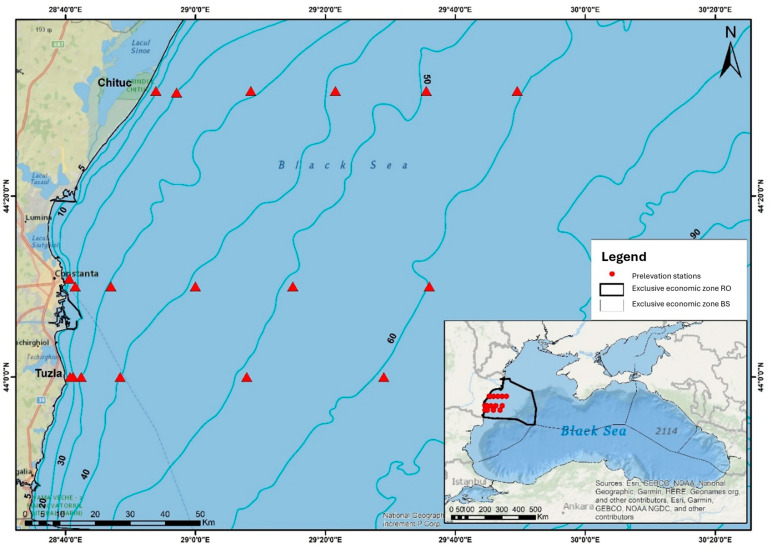
Marine copepod sampling stations in the coastal zones of the Black Sea, along three transects (Chituc, Constanta, and Tuzla), from 1956 to 2015. Triangle symbols represent sampling stations.

The long-term dataset analyzed in this study was collected from the Romanian continental shelf of the Black Sea, between 1956 and 2015. A total of 490 samples, obtained from 18 stations (Figure 1), were distributed along three transects (Chituc, Constanta, and Tuzla). Sampling expeditions were conducted during both the cold periods (November–April) [2] and the warm periods (May–October) [2,40], hereafter named winter and summer seasons, respectively. Sampling within the warm and cold periods was not uniformly distributed across all months during the 1956–2015 period, due in part to logistical and financial constraints. Consequently, analyses were conducted at the seasonal level rather than the monthly level to ensure comparability across years and to account for interannual variation in sampling timing.

During the period 1956–2015, samples were collected from sampling stations distributed along three transects on the Romanian continental shelf, with station depths ranging between 10 and 60 m according to their shelf position, using Baskakova and Juday-type zooplankton nets with a 0.1 m^2^ opening and 150 μm mesh size [35]. Nets were towed vertically through the entire water column. The samples were preserved with 4% formaldehyde and analyzed under a microscope. The data used in this study originates from the database of the National Institute for Marine Research and Development “Grigore Antipa”, Constanta. Zooplankton data were provided by the Department of Marine Ecology and Biology, available on the EurOBIS platform (https://ipt.vliz.be/eurobis/, accessed on 15 November 2025). Physicochemical parameters were provided by the Department of Chemical Oceanography and Marine Pollution. Taxonomic identification and species determination were based on standardised taxonomic keys and reference guides [41,42], and classification followed by the World Register of Marine Species (WoRMS). Total density (ind/m^3^) was calculated following standard quantitative zooplankton procedures. For each sample, individuals were counted until at least 100 organisms were identified to ensure statistical reliability. In cases of very low abundance, all individuals present in the subsample were counted. The counted organisms were then extrapolated to the total sample volume and standardised by the volume of filtered water, calculated from the net mouth area and depth. Biomass (mg/m^3^, wet weight) was estimated based on average body weight tables for Black Sea zooplankton [35,43]. Species richness, community composition, density, and biomass data were analyzed across the entire water column.

The explanatory variables used in this study were measured exclusively at a depth of 10 m. They included temperature, salinity, dissolved oxygen, and concentrations of inorganic nutrients (phosphate, nitrite, nitrate, and ammonium). For physicochemical analysis, seawater samples were collected using 1 to 5 L of Nansen cylinders [44]. Environmental variables were not integrated over the full water column because long-term physicochemical data were consistently available only at 10 m depth throughout the 1956–2015 period. To ensure temporal comparability across the six decades, analyses therefore focused on this depth, acknowledging that this approach represents a simplification of vertical variability in the water column.

Temperature and salinity were measured using a reversible or conventional thermometer [44], and standardized titration techniques, respectively [3,45]. Since 2013, the CastAway CTD multi-parameter probe (SonTek CastAway CTD, San Diego, CA, USA) has been used, which integrates sensors for temperature, salinity, depth, and GPS, allowing detailed vertical profiles to be obtained. The dissolved oxygen concentrations were determined using the Winkler titration method, a protocol recognized for its accuracy in water quality assessments. Dissolved oxygen was determined following standard Winkler titration procedures [3].

Nitrate concentrations were measured following Mullin and Riley (1955) [45,46], ammonium using the indophenol blue method [3,45], and phosphate according to standard molybdenum blue procedures [3,45]. Full methodological details are available in the cited references.

### 2.2. Data Analysis and Interpretation

The map showing the sampling sites in the territorial waters of the Black Sea was generated using ArcGIS 10.4. Visualization of copepod density and biomass data was performed using boxplot diagrams created with the ggplot2 package in R (RStudio version 2025.05.0+496).

To explore differences in copepod community composition between seasons, non-metric multidimensional scaling (NMDS) was applied using the Bray–Curtis dissimilarity index. NMDS analyses were conducted in R using the metaMDS function from the vegan package. Seasonal differences in community structure were tested using permutational multivariate analysis of variance (PERMANOVA), implemented with the adonis2() function (999 permutations), based on Bray–Curtis distance matrices [47].

Similarities and differences in qualitative community composition between the warm and cold seasons were further assessed using SIMPER (Similarity Percentages) analysis in PRIMER 7 (version 7.0.24), which quantified the contribution of individual species to within-group similarity and between-group dissimilarity.

To illustrate spatio-temporal patterns in species density and biomass, shade plots were generated separately for the warm (May–October) and cold seasons (November–April) using log_10_-transformed mean densities (ind/m^3^) and biomass values (mg/m^3^). For this purpose, observations were pooled by species, transect, and fixed sampling station depth, and arithmetic means were calculated across the entire 1956–2015 period. These shade plots, therefore represent season-specific long-term mean (“climatological”) spatial patterns and are not intended to depict temporal changes over the study period.

The percentage frequency of spatial occurrence (F) was calculated by treating each sampling station as a single spatial unit. Repeated observations at the same station across years were pooled, and a species was considered present if recorded at least once at that station during a given season. Species were classified as constant (F > 50%), accessory (25% ≤ F ≤ 50%), or accidental (F < 25%), following established criteria [48,49].

$$F=\frac{n}{N}\times 100$$

F = frequency of spatial occurrence (%);
n = the number of stations where the species was present that season;
N = the total number of stations in that season.

Temporal and seasonal variability in copepod density and biomass was evaluated using one-way ANOVA, conducted separately for warm and cold seasons and among transects. Post hoc comparisons were performed using Tukey’s HSD test. Assumptions of normality and homogeneity of variance were verified using Shapiro–Wilk tests, Q–Q plots, and Levene’s tests. When necessary, data were log (x + 1) transformed.

To explore nonlinear temporal patterns, Locally Estimated Scatterplot Smoothing (LOESS) was applied for exploratory visualization, while Generalized Additive Models (GAMs) were used to formally assess relationships between copepod density or biomass and environmental variables. LOESS [50] and GAM [51] fits were implemented using the geom_smooth() function in ggplot2. The combination of these methods provides complementary insights, as LOESS highlights general tendencies and GAM quantifies their ecological drivers, a dual approach often used in marine ecological analyses [52]. Environmental GAM analyses were restricted to the Constanta transect, which provided the most complete and temporally consistent physicochemical dataset.

For transect-specific temporal analyses, copepod density and biomass were averaged across all stations within each transect for each year and season before assessing long-term trends. Comparisons among stations located at different bottom depths refer to fixed sampling station depths on the continental shelf (10–60 m) and not to vertical strata within individual stations.

Because of the long temporal extent and heterogeneous sampling design, different analytical objectives required different data pooling strategies. Pooling was therefore adapted to the purpose of each analysis, and all aggregation levels are explicitly defined to ensure transparency and reproducibility (Table S1).

Analyses were restricted to data collected up to 2015, as only datasets covering this period have been fully validated, standardized, and archived in publicly accessible repositories, ensuring consistency and reproducibility across the six-decade time series.

## 3. Results

### 3.1. Copepods Community Structure

Between 1956 and 2015, a total of 23 species of copepods were observed, representing 13 families belonging to Calanoida and Cyclopoida orders (Table S2). Species-level identification of Harpacticoida was not carried out over time.

The SIMPER analysis showed that the dissimilarity between the seasons (warm and cold) was higher (63.5%) than the similarity within each season (warm 42.0%; cold 34.4%) (Table 1). The most important species that explained the differences between copepod communities were *Oithona nana*, *A. clausii*, *P. elongatus*, and *P. parvus parvus*, which collectively contributed more than 68% to the dissimilarity between the two periods. In addition, *Centropages kroyeri* contributed exclusively to the structure of the warm season.

The SIMPER analysis was performed using all stations and samples across the entire study area for each season, treating each station-year combination as an independent observation. This approach allows the analysis to capture both spatial and temporal variability in copepod communities. Consequently, the reported dissimilarities and species contributions reflect combined effects of seasonal, spatial, and interannual differences, providing a robust overview of community changes between warm and cold periods.

**Figure 2:**
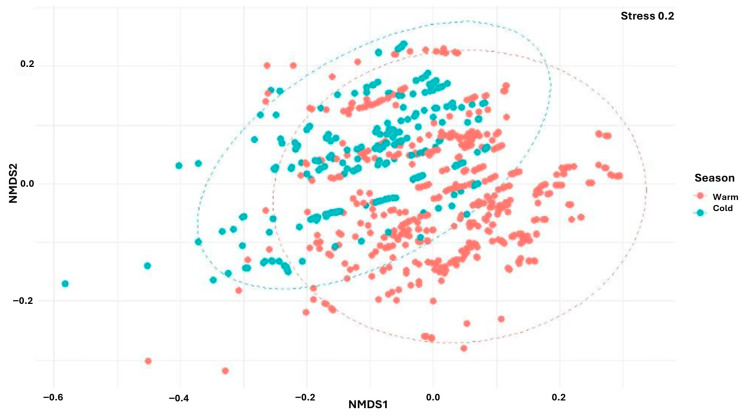
The NMDS analysis of copepod community structure, using the Bray–Curtis index (density, biomass).

The NMDS analysis of biological data clearly revealed distinct community structure in the warm and cold seasons (Figure 2). The PERMANOVA test indicated a statistically significant effect (*p* = 0.001, R^2^ = 0.0896) of the season on the ecological structure of marine copepods.

**Figure 3:**
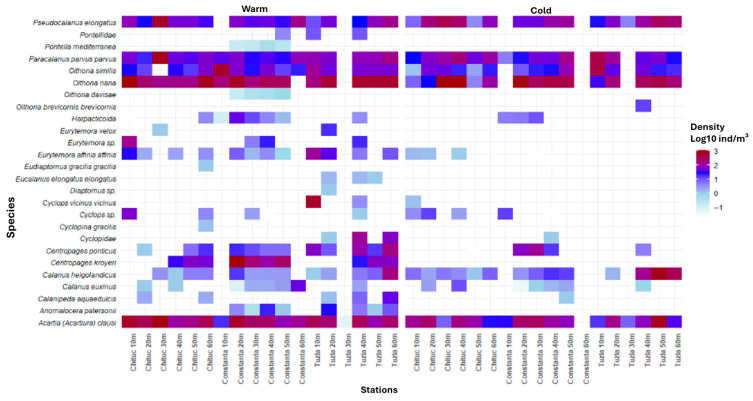
Shade plot illustrating log10-transformed density values by species and stations across seasons.

In the warm season, the highest values of densities were recorded by *O. nana* (1957, Chituc 10 m, 5492.90 ind/m^3^), *A. clausii* (1981, Chituc 20 m, 3361.08 ind/m^3^), *C. ponticus* (1967, Constanta 20 m, 1178.39 ind/m^3^), *P. parvus parvus* (1991, Chituc 30 m, 810.33 ind/m^3^), and *P. elongatus* (1991, Chituc 30 m, 653.60 ind/m^3^) (Figure 3).

In the cold season, the highest density values were recorded for *O. nana* (1957, Chituc 40 m, 2860.23 ind/m^3^), *O. similis* (1959, Constanta 50 m, 1778.76 ind/m^3^), *A. clausii* (1986, Constanta 30 m, 1760.71 ind/m^3^), *C. helgolandicus* (1957, Tuzla 50 m, 1583.48 ind/m^3^), and *P. parvus parvus* (1981, Tuzla 10 m, 1242.86 ind/m^3^) (Figure 3).

**Figure 4:**
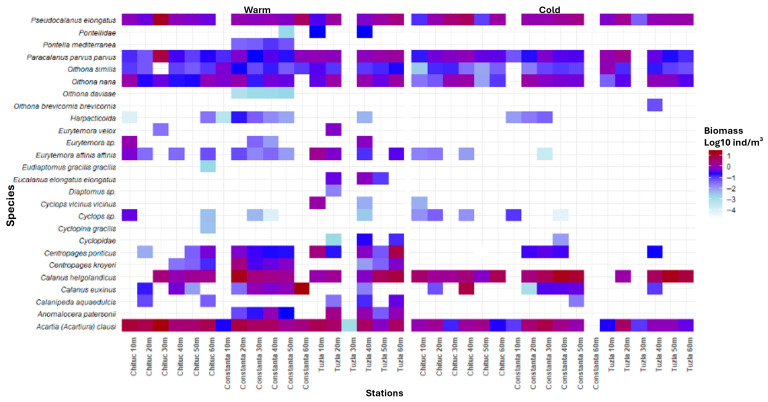
Shade plot illustrating log10-transformed biomass values by species and stations across seasons.

In the warm season, the highest annual average biomass values were recorded for *A. clausii* (1981, Chituc 20 m, 75.83 mg/m^3^), *C. helgolandicus helgolandicus* (1956, Tuzla 50 m, 40.37 mg/m^3^), *C. euxinus* (2010, Constanta 60 m, 31.86 75.83 mg/m^3^), *P. elongatus* (1991, Chituc 30 m, 17.99 mg/m^3^), and *C. ponticus* (1986, Tuzla 60 m, 16.92 mg/m^3^) (Figure 4).

In the cold season, the highest biomass values were recorded by *C. helgolandicus helgolandicus* (1991, Constanta 40 m, 57.94 mg/m^3^), *A. clausii* (1986, Constanta 30 m, 44.91 mg/m^3^), *C. euxinus* (1997, Chituc 40 m, 16.89 mg/m^3^), *P. elongatus* (1997, Chituc 40 m, 15.06 mg/m^3^), and *O. nana* (1957, Chituc 40 m, 4.14 mg/m^3^) (Figure 4).

In the warm season, out of 22 species (Table S3), 10 were constant (frequency ≥ 50%)—*A. clausii*, *P. parvus parvus*, *O. nana*, *O. similis*, *P. elongatus*, *C. helgolandicus helgolandicus*, *C. ponticus*, *Eurytemora affinis affinis*, *C. kroyeri*, and *C. euxinus*, three were accessories (25–49%)—*Anomalocera patersonii*, *Calanipeda aquaedulcis*, and *Cyclops* sp., and nine species were accidental (1–24%)—*Eurytemora* sp., *Oithona davisae*, *Pontella mediterranea*, *Eucalanus elongatus elongatus*, *Cyclops vicinus vicinus*, *Eurytemora velox*, *Cyclopina gracilis*, *Diaptomus* sp., and *Eudiaptomus gracilis gracilis*.

In the cold season, out of 13 species (Table S3), six were classified as constant: *A. clausi*, *P. parvus parvus*, *O. nana*, *O. similis*, *P. elongatus*, and *C. helgolandicus helgolandicus.* Three species were classified as accessories: *C. ponticus*, *C. euxinus*, and *Cyclops* sp., and four species were classified as accidental: *E.a. affinis*, *C. aquaedulcis*, *C. vicinus vicinus*, and *Oithona brevicornis brevicornis.*

### 3.2. Long-Term Dynamics of Copepod Communities

During the warm period, the highest annual average density (1008.0 ind/m^3^) was recorded in 1975, and the lowest (0.4 ind/m^3^) in 2002 (Figure S1). The maximum annual biomass average (14.78 mg/m^3^) was recorded in 1981, and the lowest in 2002 (0.007 mg/m^3^) (Figure S2).

The highest average annual density (755.8 ind/m^3^) during the cold season was recorded in 1959, and the lowest (0.48 ind/m^3^) in 2002 (Figure S1), while the highest average annual biomass (5.25 mg/m^3^) was recorded in 1957, and the lowest (0.006 mg/m^3^) in 2002 (Figure S2).

**Figure 5:**
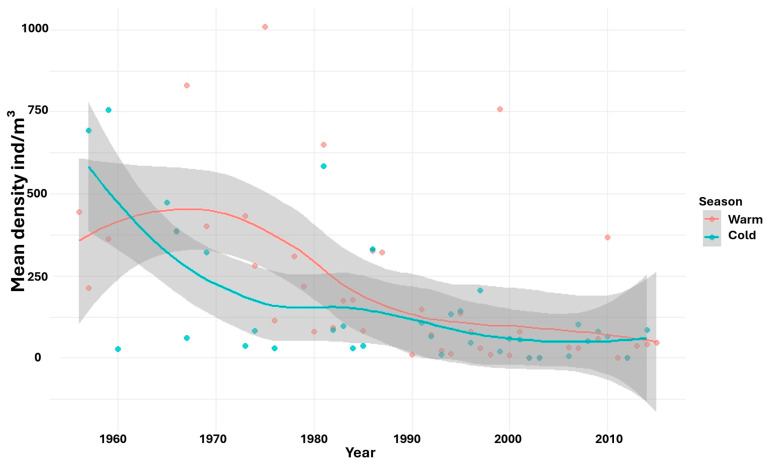
LOESS regression illustrates non-linear temporal dynamics of copepod mean density by year and season. For each year and season, densities were spatially averaged across all sampling stations. Shaded areas represent the 95% confidence intervals associated with the LOESS fits.

**Figure 6:**
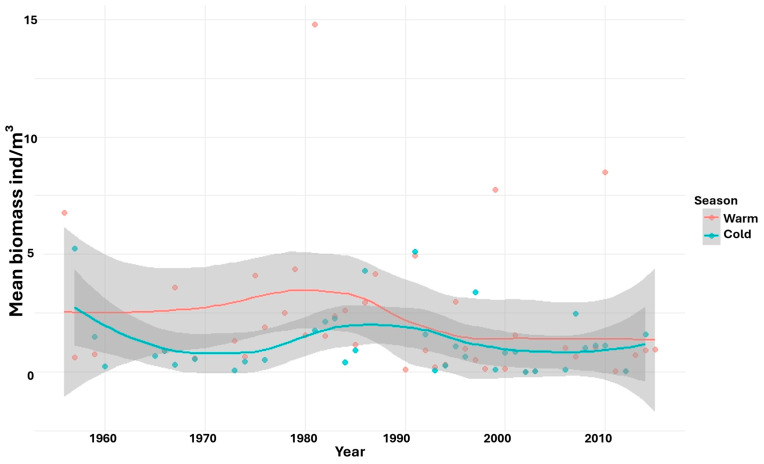
LOESS regression illustrates non-linear temporal dynamics of mean copepod biomass by year and season. For each year and season, biomass values were spatially averaged across all sampling stations. Shaded areas represent the 95% confidence intervals associated with the LOESS fits.

At the interannual scale, statistically significant differences in copepod density were observed in both the warm (F = 4.29, *p* < 2 × 10^−16^) and cold (F = 4.32, *p* = 5.17 × 10^−14^) seasons. A notable decline in density occurred during the 1960s in the cold season, whereas in the warm season, a pronounced decrease was recorded during the 1980s (Figure 5). Biomass also exhibited interannual variation, but statistically significant differences were detected only in the warm season (F = 4.35, *p* = 2 × 10^−16^), with a marked decline observed during the 1980s (Figure 6).

### 3.3. Spatial Variability of Long-Term Copepod Dynamics Across Transects and Depths

GAM analysis revealed spatial differences in the long-term trends of copepod density and biomass along the studied transects, indicating that the magnitude and direction of temporal changes vary between locations. This highlights not only the overall spatial distribution of copepods but also how trends in their abundance and biomass differ across transects, reflecting the influence of local environmental conditions, hydrography, and nutrient availability.

**Figure 7:**
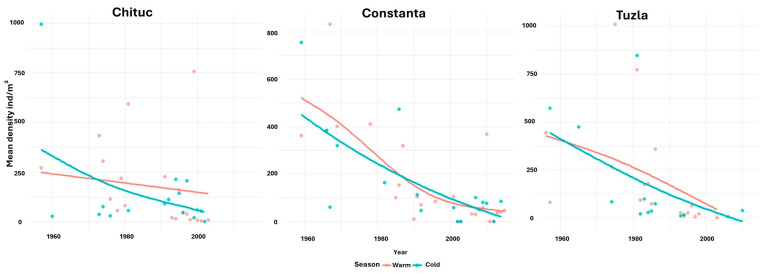
GAM showing copepod density dynamics over time across the three transects (Chituc, Constanta, and Tuzla). Vertical axis limits differ among panels to enhance the visualization of within-transect temporal variability.

Based on transect-averaged values, copepod density exhibited a consistent and gradual temporal decline over the study period at the Chituc transect, with a more pronounced decrease during the cold season. The rate of decline became less steep from the mid-1970s onward. At the Constanta transect, temporal trends showed a steeper overall decrease in copepod density, with only minor seasonal differences; however, after the mid-1980s, the downward trend weakened during the warm season. At the Tuzla transect, copepod density also displayed a continuous long-term decline, with limited seasonal variability and slightly higher mean values during the warm season (Figure 7).

To examine differences among sampling stations located at different bottom depths, copepod abundance was analyzed using seasonal mean values calculated for each station depth (10–60 m) by pooling observations across the entire 1956–2015 period.

Overall, copepod abundance tended to be higher at stations located at 20–30 m and decreased toward stations situated at greater depths (50–60 m) during the warm season. In contrast, densities were generally lower across all station depths during the cold season. However, season-specific patterns were evident at stations located at 40–50 m along the Chituc and Tuzla transects and between 30 and 50 m along the Constanta transect, where copepod abundances were relatively higher than expected during the cold season.

**Figure 8:**
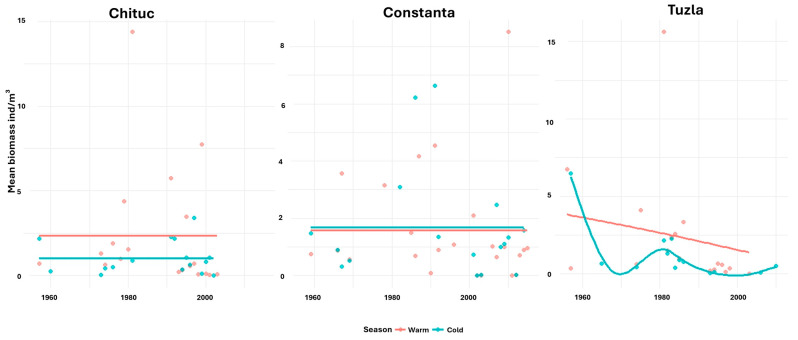
GAM showing copepod biomass dynamics over time across the three transects (Chituc, Constanta, and Tuzla). Vertical axis limits differ among panels to enhance the visualization of within-transect temporal variability.

Temporal trends of copepod biomass revealed distinct patterns among transects. At Chituc, copepod biomass showed moderate variability throughout the study period, with slightly higher values during the warm season. A similar trend was observed at Constanta, where biomass remained relatively constant. In contrast, at Tuzla, biomass declined steadily during the warm season, while in the cold season it showed a steep decrease in the early decades, followed by a tendency toward recovery during the late 1970s and early 1980s, and then a gradual decline thereafter (Figure 8).

During the warm season, copepod biomass decreased significantly at sampling stations located at greater bottom depths, with the lowest values recorded at stations situated at 40–60 m in the Chituc transect, 30–50 m in the Constanta transect, and across the 10–60 m depth range in the Tuzla transect (*p* < 0.05), except at the 30 m station in Chituc, where a significant increase was observed (*p* = 0.018). Conversely, during the cold season, higher biomass values were recorded at stations located at 40 m in Chituc, 30–50 m in Constanta, and 50 m in Tuzla (*p* < 0.05).

### 3.4. Environmental Drivers of Copepod Communities

The GAM for copepod density explained 28.1% of the variability (adjusted R^2^ = 0.251). Seasonal analyses revealed distinct drivers: in the warm season, density was significantly influenced by phosphates (F = 20.34, *p* < 2 × 10^−16^), and nitrites (F = 2.515, *p* = 0.04396), and marginally by nitrates (F = 2.194, *p* = 0.06959). In the cold season, density correlated with temperature (F = 3.039, *p* = 0.01165), salinity (F = 9.648, *p* = 0.00194), nitrites (F = 10.641, *p* = 0.00114), and ammonium (F = 5.314, *p* = 0.00069) (Table 2). The results indicate seasonally differentiated controls over copepod density.

For copepod biomass (mg/m^3^), the GAM explained 20.8% of the total deviance (adjusted R^2^ = 0.179). In the warm season, biomass was driven solely by phosphates (F = 16.182, *p* < 2 × 10^−16^), whereas in the cold season, both phosphates (F = 4.532, *p* = 5.55 × 10^−5^) and ammonium (F = 9.200, *p* < 2 × 10^−16^) had significant effects, with salinity showing a marginal influence (F = 2.765, *p* = 0.0967) (Table 3). These results highlight seasonal shifts in the abiotic factors controlling copepod density and biomass.

The GAM analysis revealed seasonally differentiated effects of environmental predictors on copepod density and biomass. During the warm season, phosphate had a strong positive effect, supporting higher density and biomass, while temperature also contributed positively, and other nutrients had weaker or inconsistent effects. In the cold season, temperature and regenerated nitrogen (ammonium, nitrites) showed positive influences, whereas salinity exerted a generally negative effect, reflecting sensitivity to offshore conditions. Overall, nutrient-driven controls dominate in the warm season, whereas physical and chemical factors play a stronger role during the cold season.

## 4. Discussion

This study reveals pronounced long-term changes in copepod communities along the Romanian Black Sea coast. Analysis of one of the most extensive multi-decadal datasets demonstrates a consistent decline in copepod abundance, shifts in species composition, and marked seasonal and spatial variability. Temperature, salinity, oxygen, and nutrient concentrations emerged as key drivers shaping these patterns, indicating strong links between copepod dynamics and environmental conditions. The findings highlight the sensitivity of copepod communities to both natural variability and anthropogenic pressures, particularly eutrophication, and underscore the importance of long-term monitoring for detecting ecosystem change.

### 4.1. Species Community Structure

The long-term analysis of copepod communities along the Romanian Black Sea coast (1956–2015) reveals a pronounced restructuring of species composition driven by interdecadal variability and strong seasonal contrasts. A total of 23 copepod species were recorded cumulatively over the entire study period; however, species richness at the seasonal scale was substantially lower. Cold-season communities comprised only 13 species, indicating a marked contraction of diversity, particularly after the late 1970s, when several sensitive species began to decline.

During the warm season, copepod biomass exhibited pronounced interannual variability, with peak values occurring in different years for the main taxa: *A. clausii* in 1981, *C. helgolandicus helgolandicus* in 1956, and *C. euxinus* in 2010. Based on frequency of spatial occurrence within the study area, the warm-season assemblage comprised 10 constant species (≥50% occurrence; e.g., *A. clausii*, *P. parvus parvus*, *O. nana*), three accessory species (25–49%; *A. patersonii*, *C. aquaedulcis*, *Cyclops* sp.), and nine accidental species (1–24%; e.g., *Eurytemora* sp., *O. davisae*, *P. mediterranea*). *O. davisae* was discovered during regular monitoring along the western coast of the Black Sea in 2009–2012, contributing significantly to secondary production and being an important component of marine trophic chains, presenting a food source for planktivorous fish and many predatory fish larvae [53].

In the cold season, maximum biomass values were observed in 1991 for *C. helgolandicus helgolandicus*, 1986 for *A. clausii*, and 1997 for *C. euxinus*. The cold-season community showed a reduced number of constant species (six taxa, including *A. clausii*, *P. parvus parvus*, *O. nana*), alongside three accessory species (*C. ponticus*, *C. euxinus*, *Cyclops* sp.) and four accidental species (e.g., *E. affinis affinis*, *C. aquaedulcis*, *C. vicinus vicinus*). These results indicate a season-dependent restructuring of copepod assemblages, with lower taxonomic stability during the cold period.

Across the entire 1956–2015 period, warm-season assemblages consistently included a higher number of constant species and a greater contribution of accessory and accidental taxa, indicating increased species richness and compositional heterogeneity compared to the cold season.

SIMPER results identify *O. nana*, *A. clausii*, *P. elongatus*, and *P. parvus parvus* as the major contributors to seasonal dissimilarity, accounting for over 68% of warm–cold season separation. *C. kroyeri* acts as a strong indicator of warm-season conditions, while larger calanoids, common in the 1960s–1970s, have become infrequent in recent decades, reflecting sensitivity to warming, nutrient shifts, and stratification changes [2,18,19,54].

Long-term trends in abundance and biomass reinforce this restructuring. The 1960s (cold season) and 1980s (warm season) exhibited pronounced declines, with a system-wide collapse reaching minimum values in 2002, suggesting cumulative impacts from eutrophication, climatic variability, and hydrological instability. This period coincides with documented ecosystem disturbances in the Black Sea, including anomalous climatic conditions and increased top-down pressure from gelatinous predators, particularly *M. leidyi*, which are known to strongly suppress copepod populations [22,38,55,56,57]; thus, the 2002 minimum likely reflects a real ecological signal. These shifts coincide with the increasing dominance of small-bodied, fast-reproducing species such as *O. nana* and *A. clausi*, which thrived particularly after the 1990s, marking a transition toward an opportunistic, resilience-oriented community typical of stressed coastal ecosystems [2,18,19,54].

Overall, the copepod assemblage of the Romanian Black Sea has shifted from a historically more diverse, calanoid-rich community (1956–1975) to a simplified structure increasingly dominated by a few highly tolerant species (1990–2015). This transformation—expressed both seasonally and across the six decades—highlights the vulnerability of zooplankton communities to long-term environmental pressures and underscores the need for continued species-level monitoring to detect and understand ecological change.

Although certain copepod taxa exhibit apparent resilience by maintaining their presence under changing environmental conditions, this taxon-level persistence does not necessarily imply resilience of the broader pelagic ecosystem. A shift toward smaller-bodied, opportunistic copepods is often associated with reduced trophic efficiency and diminished energy transfer to higher trophic levels, leading to lower food quantity and quality for planktivorous fish and their early life stages [24,58]. Consequently, the observed restructuring of copepod communities may reflect a loss of ecosystem-level resilience, despite the apparent stability of specific taxa, with potential implications for fish recruitment and food-web functioning [22].

The seasonal shift in dominant environmental drivers likely reflects underlying mechanisms controlling copepod dynamics. During the warm season, phosphate acts as a key driver because it likely represents a limiting nutrient for phytoplankton under stratified conditions, indirectly enhancing copepod density and biomass through increased food availability and quality [24,59]. In contrast, during the cold season, primary production is reduced, and copepod populations are more strongly influenced by physical conditions. Temperature regulates metabolic rates, development, and overwintering survival, while salinity reflects water-mass structure and mixing, determining habitat stability and vertical distribution [21,24]. These mechanisms explain why nutrient-driven controls dominate in summer, whereas physical drivers govern cold-season dynamics.

### 4.2. Long-Term Temporal and Spatial Patterns of Copepod Communities

Over the entire period from 1956 to 2015, copepod density showed a marked and continuous decline. The highest values were recorded in the 1960s, followed by a progressive decrease in the subsequent decades, which reached low and stable levels after 2000.

The pronounced decline in copepod density and biomass during the 1980s coincided with the collapse of pelagic food webs driven by multiple stressors, including nutrient enrichment, hypoxia, overfishing, and the introduction of the invasive ctenophore *Mnemiopsis leidyi* [55]. This gelatinous predator exerted strong top-down control over mesozooplankton populations, particularly calanoid copepods such as *A. clausii*, *P. parvus parvus*, and *P. elongatus*, which previously dominated coastal pelagic assemblages. The combined impact of predation pressure and reduced food quality likely caused the drastic decline in copepod stocks observed until 2002.

Seasonal differences, evidently at the beginning of the period, gradually diminished. Biomass dynamics indicated a long-term declining trend, with peak values reached in the 1980s, followed by a gradual reduction in both warm and cold seasons. Biomass was generally higher during the warm season, but the seasonal difference decreased over time. The downward trend observed in recent decades suggests significant changes in ecosystem productivity.

GAM analysis revealed pronounced spatial and seasonal variability in copepod density and biomass among the Chituc, Constanta, and Tuzla transects, reflecting differences in the spatio-temporal organization of zooplankton communities driven by seasonal stratification, local hydrographic conditions, and productivity regimes. Seasonal patterns showed higher densities and biomass during the warm season, corresponding to elevated temperatures and enhanced primary productivity, whereas the cold season was characterized by lower abundances associated with reduced phytoplankton availability and thermal constraints [1,2,25,54,60]. During the warm season, maximum densities were consistently observed at intermediate depths (20–30 m) across transects, likely reflecting higher food availability above the thermocline [61,62]. In contrast, during the cold season, higher densities were generally recorded at deeper stations (30–50 m), suggesting vertical redistribution of copepods toward more stable, oxygen-rich waters, a strategy commonly reported for calanoid copepods such as *P. elongatus*.

The Chituc transect, which is strongly influenced by the Danube outflow, exhibited moderate copepod density and biomass variability until the 1980s, followed by a gradual long-term decline (Figure 7 and Figure 8). This temporal pattern coincides with changes in river discharge and associated nutrient regimes, which are known to affect local productivity and zooplankton community structure. Similar responses to freshwater input and nutrient enrichment have been reported in previous studies from the Black Sea and other river-influenced coastal systems [2,24,54,63,64].

At the Constanta transect, both copepod density and biomass showed a more continuous and pronounced decline over time (Figure 7 and Figure 8), with limited seasonal contrast, consistent with stronger and persistent coastal pressures. These trends correspond to areas affected by urbanization, port activities, and coastal development, suggesting a cumulative impact of human-induced changes in nutrient input, salinity, and local circulation on copepod abundance and community composition [36,65], as also documented in earlier studies from the Romanian Black Sea coast [2,24,54,63,64].

In contrast, the Tuzla transect displayed a continuous decline in copepod abundance, with only slightly higher densities during the warm season and a sharp reduction during the cold season (Figure 7 and Figure 8). This pattern is consistent with the stronger influence of offshore marine waters, where copepod variability is largely governed by basin-scale circulation, deep-water processes, and seasonal hydrographic dynamics rather than direct coastal forcing [2,66,67]. Overall, these transect-specific temporal patterns demonstrate that, although seasonal redistribution of copepods is a common adaptive response, long-term changes in density and biomass are modulated by spatial gradients in salinity, nutrient availability, and hydrodynamic stability, highlighting the sensitivity of copepod communities to both environmental variability and anthropogenic pressures (Figure 5, Figure 6, Figure 7 and Figure 8) [61,62,68,69].

Although all transects are influenced by the Danube at the basin scale, their local hydrographic regimes differ along the Romanian coast as a result of spatial gradients in freshwater input, salinity, nutrient availability, and hydrodynamic stability, leading to distinct ecological responses. The Chituc transect, located in the northern sector, is more persistently affected by riverine inputs and associated nutrient enrichment, resulting in lower salinity and higher temporal variability, conditions known to influence zooplankton distribution and abundance [54,70,71]. In contrast, the Constanta transect reflects the combined influence of Danube-derived signals and intense local anthropogenic pressures related to urban discharge, port activities, and coastal modification, which have been linked to pronounced changes in mesozooplankton density and community structure [63]. The Tuzla transect, situated further south, is comparatively more influenced by offshore marine waters and alongshore circulation, exhibiting weaker riverine signatures, higher salinity stability, and more uniform seasonal dynamics, consistent with circulation patterns described for the western Black Sea shelf [66]. Together, these documented transect-specific differences in abiotic forcing provide a mechanistic framework for interpreting the spatial variability in copepod density and biomass observed in this study.

During the warm season, elevated temperatures, enhanced primary productivity, and abundant food resources sustain higher densities, with species such as *A. clausii* and *O. similis* dominating shallow, nutrient-rich waters [2,25,54,58,62]. In contrast, cold-season biomass is primarily regulated by vertical mixing and nutrient redistribution, which can lead to increased densities at deeper stations despite lower surface productivity [1,60,62]. Overall, the distribution of copepods is shaped by the interplay of thermal stratification, nutrient availability, and oxygen conditions, with communities responding sensitively to local hydrographic gradients, consistent with transect-specific patterns observed in previous studies in the Black Sea [70,71,72].

### 4.3. Environmental Drivers of Copepod Communities

Long-term trends indicate that eutrophication has been a major anthropogenic driver of copepod community dynamics along the Romanian coast. Nutrient inputs (N and P) from the Danube River and urban/agricultural sources intensified during the 1970s–1990s, leading to recurrent phytoplankton blooms, diel oxygen fluctuations, hypoxia, and altering the plankton structure of pelagic food webs [3,26,39,73].

Generalized additive models (GAM) revealed that copepod density and biomass in the Romanian Black Sea are shaped by seasonally distinct physicochemical drivers, explaining 28.1% of density and 20.8% of biomass variability. In the warm season, both density and biomass were primarily controlled by phosphate concentrations, with additional contributions from nitrites and, to a lesser extent, nitrates, reflecting the role of nutrient enrichment in stimulating phytoplankton growth and supporting copepods such as *A. clausii* and *P. parvus parvus* [71,72]. In contrast, cold-season dynamics were more strongly influenced by hydrographic variables such as temperature and salinity, alongside nitrite and ammonium concentrations. These parameters regulate stratification, vertical mixing, and overwintering behavior, while recycled nitrogen sustains basal production under low-chlorophyll conditions [74,75,76]. Phosphate remained a consistent driver for biomass across seasons, with ammonium and salinity effects in winter highlighting the importance of regenerated nutrients and water mass exchanges in maintaining copepod populations [74,75,76]. On the Bulgarian coast, community shifts were linked to eutrophication and invasive species [31], and overall, the Romanian Black Sea coast exhibits long-term trends shaped by both climatic variability and anthropogenic pressures, consistent with regional studies [2,25,31].

Overall, these results indicate a seasonal shift in the dominant controls of copepod productivity. During the warm season, when the water column is stratified, nutrient availability, particularly phosphate, is decoupled from physical mixing and acts as a key driver of primary production, indirectly supporting higher copepod density and biomass. In contrast, during the cold season, when the water column is well mixed, copepod persistence is largely controlled by hydrographic conditions (temperature and salinity) and nitrogen regeneration, which together maintain background productivity and habitat suitability (Table 4).

The moderate explanatory power of the models (GAM, explaining 28.1% of density and 20.8% of biomass variability) suggests that additional factors, such as predation, competition, and mesoscale circulation, also contribute to community structure. These findings emphasize the combined importance of bottom-up and top-down controls in understanding and managing Black Sea pelagic ecosystems in line with the Marine Strategy Framework Directive (MSFD) [36,37,65]. In addition to bottom-up controls, copepod dynamics are likely shaped by both top-down and lateral processes. Among top-down drivers, predation by planktivorous fish, especially in early life stages, can selectively reduce the abundance of larger, energy-rich copepods [22,58]. Gelatinous predators, particularly ctenophores such as *M. leidyi*, also exert strong top-down control by consuming copepods and competing for shared food resources [85,86]. Lateral processes, including advection and alongshore transport, redistribute plankton assemblages across transects, especially in coastal areas affected by river plumes and seasonal circulation [66]. Together, these mechanisms likely explain additional variability in copepod density and biomass beyond the bottom-up drivers captured by the GAMs.

Although this study presents a comprehensive long-term assessment of copepod communities in the Romanian Black Sea, certain limitations must be acknowledged. First, sampling coverage and frequency were not fully consistent throughout the six decades, which may have introduced methodological variability in addition to ecological change. Second, despite the breadth of the dataset, it is not continuous: some years and seasons are underrepresented, which constrains the ability to fully capture interannual variability.

Nevertheless, the combination of historical and recent observations provides unique and valuable insights into the long-term dynamics of copepods, offering a crucial perspective on biodiversity shifts and ecosystem responses in the northwestern Black Sea.

### 4.4. Copepod Density and Biomass in Other Marine Ecosystems

Long-term declines in copepod density and biomass, together with shifts toward smaller-bodied and opportunistic species, have been widely reported in coastal and semi-enclosed marine ecosystems worldwide. In the Mediterranean Sea, long-term observations document reductions in large calanoid copepods, increased dominance of cyclopoids, and simplified community structures, patterns commonly attributed to ocean warming, enhanced water-column stratification, and changes in phytoplankton size structure and food quality [22,24,74]. Similar trends have been reported in eutrophic and river-influenced coastal systems such as the Mediterranean Sea, the Baltic Sea, and North Sea coastal zones, as well as other temperate shelf ecosystems, where nutrient enrichment and climate-driven variability favor fast-reproducing copepod taxa and lead to reduced trophic transfer efficiency [19,23].

Comparable copepod responses have also been observed in transitional and temperate coastal ecosystems, including estuarine and lagoonal environments, where long-term nutrient enrichment and warming promote community simplification and increased dominance of small omnivorous species, often accompanied by enhanced interannual variability in abundance and biomass [74]. Together, these studies indicate that copepod communities across diverse marine regions respond in a broadly consistent manner to combined climatic and anthropogenic pressures.

Some limitations of this study should be acknowledged. Analyses of environmental drivers were restricted to the Constanta transect, which provides the most consistent long-term physicochemical time series; therefore, inferred relationships primarily reflect conditions characteristic of this sector rather than the entire Romanian shelf. In addition, temporal heterogeneity in sampling effort is inherent to multi-decadal historical datasets and may influence estimates of short-term variability. Finally, the use of environmental measurements at 10 m depth ensures temporal comparability but does not fully capture vertical processes, particularly during stratified warm-season conditions. Despite these constraints, the dataset represents one of the longest and most comprehensive copepod time series available for the Romanian Black Sea coast, allowing robust assessment of long-term ecological trends and ecosystem responses to combined climatic and anthropogenic pressures.

Future studies should extend the analysis of environmental drivers to additional transects as more harmonised long-term physicochemical datasets become available, allowing improved assessment of spatial variability along the Romanian Black Sea coast. Enhanced and more uniform sampling coverage, together with the integration of historical data and modern monitoring approaches, would strengthen the detection of interannual and decadal trends. Incorporating depth-resolved environmental measurements would further improve understanding of the role of stratification and subsurface processes in shaping copepod community dynamics, particularly during the warm season.

## 5. Conclusions

Analysis of a multi-decadal dataset reveals pronounced long-term changes in copepod communities along the Romanian Black Sea coast. Copepod abundance and biomass have declined consistently, accompanied by shifts in species composition and pronounced seasonal and spatial variability. These patterns indicate progressive alterations in the trophic structure and functioning of the northwestern Black Sea ecosystem. The observed trends are likely driven by the combined effects of eutrophication, rising temperatures, and hydrodynamic variability, which have modified nutrient availability and primary productivity over recent decades. The contrasting seasonal controls—where temperature and salinity dominate during the cold season and nutrient concentrations during the warm season—highlight the complex, season-specific environmental controls shaping copepod dynamics. Long-term shifts in copepod communities may propagate through the food web, affecting fish larvae and other planktivorous organisms, and emphasize the importance of sustained monitoring to understand and manage ecosystem change in the Black Sea.

## Figures and Tables

**Table 1:** Results of the SIMPER (Similarity Percentages) analysis based on the Bray–Curtis index between the ‘Warm’ and ‘Cold’ groups.

Groups Warm & Cold						
Average Dissimilarity = 63.48						
	Group Warm	Group Cold				
**Species**	Av.Abund	Av.Abund	Av.Diss	Diss/SD	Contrib%	Cum.%
*Oithona nana*	396.41	323.11	19.61	1.32	30.90	30.90
*Acartia (Acartiura) clausii*	292.26	154.57	12.29	1.16	19.36	50.26
*Pseudocalanus elongatus*	89.50	117.35	6.21	1.01	9.78	60.04
*Paracalanus parvus parvus*	102.58	91.72	5.37	0.76	8.45	68.49
*Centropages kroyeri*	118.34	0	5.31	0.59	8.36	76.86

Av.Abund—average abundance of the species in the group. Av.Diss—average contribution of the species to the dissimilarity. Diss/SD—ratio of the contribution to its standard deviation. Contrib%—percentage contribution of the species to total dissimilarity. Cum.%—cumulative percentage contribution.

**Table 2:** Results of the GAM relating copepod density to physicochemical predictors along the Constanta transect.

Parameter	Warm Season—*p*	Warm Season—F	Cold Season—*p*	Cold Season—F
**T°C**	0.32342	1.078	**0.01165** *	**3.039**
**Salinity**	0.54548	0.366	**0.00194** **	**9.648**
**O** **_2_**	0.66827	0.185	0.9711	0.003
**PO_4_^3−^**	**2** × 10^−16^ ***	**20.338**	0.33515	1.139
**NO_2_^−^**	0.04396 *	**2.515**	0.00114 **	**10.641**
**NO_3_^−^**	*0.06959 **· ^·^***	*2.194*	*0.05969 **· ^·^***	*2.214*
**NH_4_^+^**	0.33167	0.987	**0.00069** ***	**5.314**

Signification. codes: ‘***’ 0.001 ‘**’ 0.01 ‘*’ 0.05 ‘^·^’ 0.1 ‘·’ 1.

**Table 3:** Results of the GAM relating copepod biomass to physicochemical predictors along the Constanta transect.

Parameter	Warm Season—*p*	Warm Season—F	Cold Season—*p*	Cold Season—F
**T°C**	0.2602	1.270	0.7061	0.140
**Salinity**	0.1718	1.830	*0.0967 · ^·^*	*2.765*
**O** **_2_**	0.3402	0.911	0.2995	1.143
**PO_4_^3−^**	2 × 10^−16^ ***	**16.182**	5.55 × 10^−5^ ***	**4.532**
**NO_2_^−^**	0.2244	1.412	0.3119	1.024
**NO_3_^−^**	0.3522	0.870	0.9437	0.006
**NH_4_^+^**	0.1985	1.461	2 × 10^−16^ ***	**9.200**

Signification. codes: ‘***’ 0.05 ‘^·^’ 0.1 ‘·’ 1.

**Table 4:** Seasonal effects of environmental drivers on copepod density and biomass in the Black Sea.

Factor	Warm Season	Cold Season	References
**Temperature**	High, stimulates metabolism	Low limits growth	[77,78,79]
**Phosphate**	Strongly influences biomass and density (algae and copepods)	Significant for biomass and density, combined with ammonium	[17,59,80]
**Ammonium**	Moderate effect	Strong effect on biomass	[17,59,80]
**Nitrites**	Significant for density	Significant for density	[17]
**Nitrates**	Marginal effect	Marginal effect	[17]
**Salinity**	Minor effect	Strong influence on density/biomass	[21,81,82,83]
**Oxygen**	Minor effect	No significant effect	[58,84]

## Data Availability

The data belong to the National Institute for Marine Research and Development “Grigore Antipa” (NIMRD) and can be accessed upon request to http://www.nodc.ro/data_policy_nimrd.php (accessed on 15 November 2025) and the EurOBIS platform https://ipt.vliz.be/eurobis/ (accessed on 15 November 2025).

## Supplementary Materials

The following supporting information can be downloaded at: https://www.mdpi.com/article/10.3390/biology15020184/s1. Table S1. Summary of data pooling strategies applied across analytical methods used in this study; Table S2. List of copepod species identified on the Romanian Black Sea coast between 1956 and 2015; Table S3. Frequency of occurrence (%) and constancy categories of copepod species identified on the Romanian Black Sea coast between 1956 and 2015; Figure S1. Annual average density of copepods by year during the warm and cold seasons, 1950–2015; Figure S2. Annual average biomass of copepods by year during the warm and cold seasons, 1950–2015.
