# Supplementary material for: Environmental Drivers and Long-Term Dynamics of Copepod Communities in the Black Sea: Contrasts Between Warm and Cold Periods
Source: Biology (Basel). 2026 Jan 19;15(2):184. doi: 10.3390/biology15020184 (PMC12837191; doi:10.3390/biology15020184)
Supplement: Supplementary file 1 [file biology-15-00184-s001.zip › biology-4042669-supplementary.pdf]

Supplementary material to the article:

Environmental Drivers and Long-Term Dynamics of Copepod Communities in the Black Sea: Contrasts Between Warm and Cold Periods

George-Emanuel Harcotă [1,2], Elena Bișinicu[2*], Luminița Lazăr[2], Florin Timofte[2], and Geta Rîșnoveanu[1]

1 University of Bucharest, Doctoral School of Ecology and Sustainability, Bucharest, Romania, george-emanuel.harcota@s.unibuc.ro; geta.risnoveanu@g.unibuc.ro

2 National Institute for Marine Research and Development "Grigore Antipa" Constanța, gharcota@alpha.rmri.ro; ebisinicu@alpha.rmri.ro; llazar@alpha.rmri.ro; ftimofte@alpha.rmri.ro

*Correspondence: gharcota@alpha.rmri.ro, ebisinicu@alpha.rmri.ro

Table S1. Summary of data pooling strategies applied across analytical methods used in this study

Analysis	Pooling level/data aggregation
**Shade plots (long-term seasonal means)**	Season × sampling station depth x species; values represent season-specific long-term means pooled over the entire 1956–2015 period
**Frequency of spatial occurrence (F)**	Per station (presence/absence pooled across years for each season)
**SIMPER**	Entire study area (all transects pooled)
**NMDS**	Entire study area (all transects pooled)
**PERMANOVA**	Entire study area (all transects pooled)
**LOESS**	Spatially averaged values (across stations or transects, depending on analysis)
**GAM**	Per transect; environmental drivers Constanta transect only

Table S2. List of copepod species identified on the Romanian Black Sea coast between 1956 and 2015.

No.	Species	Author Name	Order	Family
1.	*Acartia clausii*	Giesbrecht, 1892	Calanoida	Acartiidae
2.	*Calanus euxinus*	Hulsemann, 1991	Calanoida	Calanidae
3.	*Calanus helgolandicus helgolandicus*	(Claus, 1863)	Calanoida	Calanidae
4.	*Centropages kroyeri*	Giesbrecht, 1892-1893	Calanoida	Centropagidae
5.	*Centropages ponticus*	Karavaev, 1895	Calanoida	Centropagidae
6.	*Pseudocalanus elongatus*	(Brady, 1865)	Calanoida	Clausocalanidae
7.	*Diaptomus sp.*	Westwood, 1836	Calanoida	Diaptomidae
8.	*Eudiaptomus gracilis gracilis*	(Sars G.O., 1863)	Calanoida	Diaptomidae
9.	*Eucalanus elongatus elongatus*	(Dana, 1849-1852)	Calanoida	Eucalanidae
10.	*Paracalanus parvus parvus*	(Claus, 1863)	Calanoida	Paracalanidae
11.	*Anomalocera patersonii*	Templeton, 1837	Calanoida	Pontellidae
12.	*Pontella mediterranea*	(Claus, 1863)	Calanoida	Pontellidae
13.	*Calanipeda aquaedulcis*	Krichagin, 1873	Calanoida	Pseudodiaptomidae

14.	*Eurytemora sp.*	(Poppe, 1880)	Calanoida	Temoridae
15.	*Eurytemora affinis affinis*	(Poppe, 1880)	Calanoida	Temoridae
16.	*Eurytemora velox*	(Lilljeborg, 1853)	Calanoida	Temoridae
17.	*Cyclops vicinus vicinus*	Uljanin, 1875	Cyclopoida	Cyclopidae
18.	*Cyclopina gracilis*	Claus, 1863	Cyclopoida	Cyclopinidae
19.	*Cyclops sp.*	Müller O.F., 1785	Cyclopoida	Cyclopinidae
20.	*Oithona brevicornis brevicornis*	Giesbrecht, 1891	Cyclopoida	Oithonidae
21.	*Oithona davisae*	Ferrari F.D. & Orsi, 1984	Cyclopoida	Oithonidae
22.	*Oithona nana*	Giesbrecht, 1893	Cyclopoida	Oithonidae
23.	*Oithona similis*	Claus, 1866	Cyclopoida	Oithonidae

Table S3. Frequency of occurrence (%) and constancy categories of copepod species identified on the Romanian Black Sea coast between 1956 and 2015

Species	Frequency Warm season (%)	Constancy Warm season	Frequency Cold season (%)	Constancy Cold season
Acartia (Acartiura) clausii	100	Constant	94	Constant
Paracalanus parvus parvus	94	Constant	89	Constant
Oithona nana	89	Constant	83	Constant
Oithona similis	89	Constant	83	Constant
Pseudocalanus elongatus	89	Constant	89	Constant
Calanus helgolandicus helgolandicus	72	Constant	78	Constant
Centropages ponticus	67	Constant	28	Accessory
Eurytemora affinis affinis	67	Constant	22	Accidental
Centropages kroyeri	56	Constant		
Calanus euxinus	50	Constant	39	Accessory
Anomalocera patersonii	44	Accessories		
Calanipeda aquaedulcis	28	Accessories	6	Accidental
Cyclops sp.	28	Accessories	28	Accessory
Eurytemora sp.	22	Accidental		
Oithona davisae	22	Accidental		
Pontella mediterranea	22	Accidental		
Eucalanus elongatus elongatus	17	Accidental		
Cyclops vicinus vicinus	11	Accidental	6	Accidental
Eurytemora velox	11	Accidental		
Cyclopina gracilis	6	Accidental		
Diaptomus sp.	6	Accidental		
Eudiaptomus gracilis gracilis	6	Accidental		
Oithona brevicornis brevicornis			6	Accidental

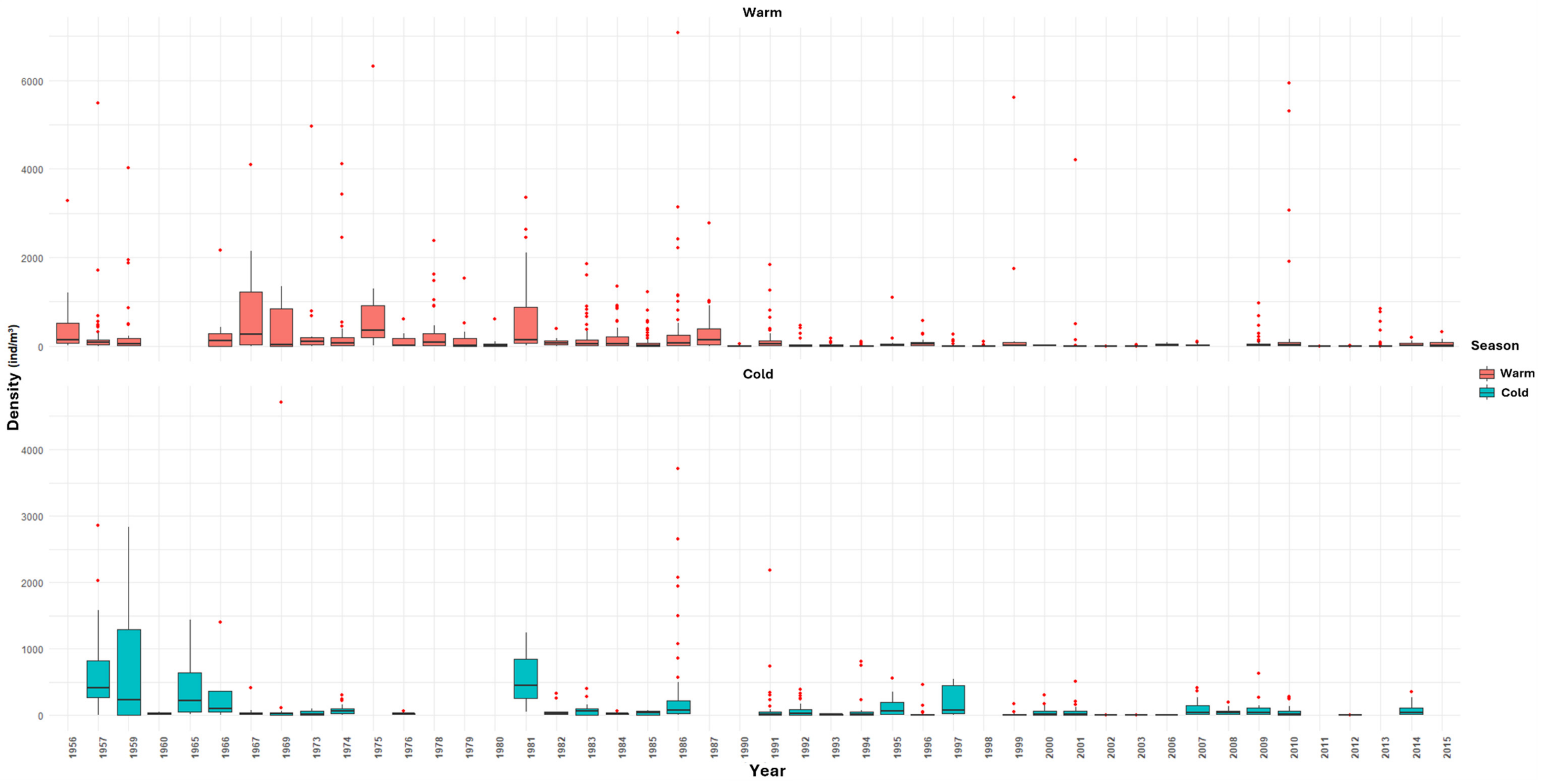

Figure S1. Annual average density of copepods by year during the warm and cold seasons, 1950–2015

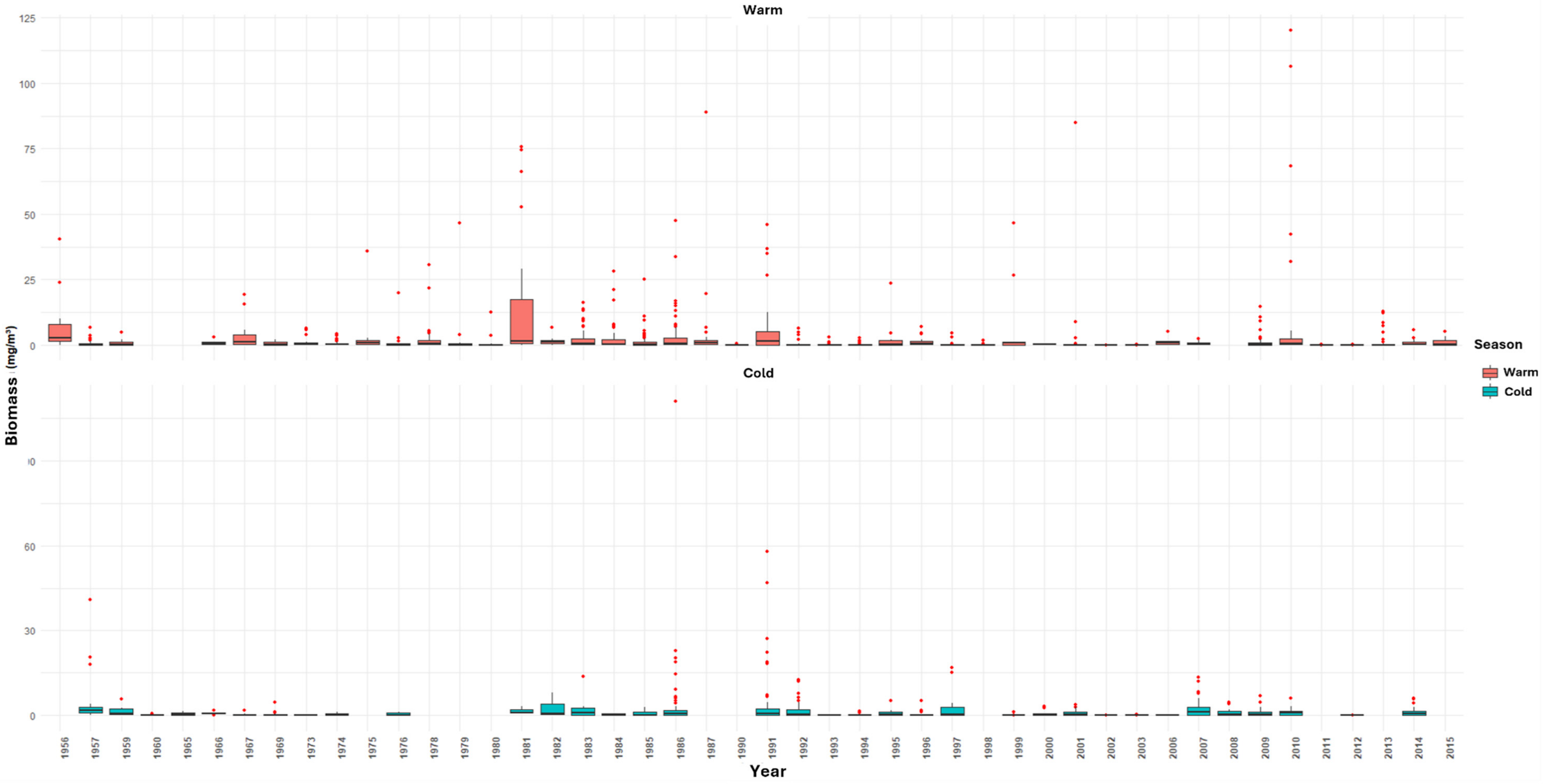

Figure S2. Annual average biomass of copepods by year during the warm and cold seasons, 1950–2015
